# FinaleToolkit: Accelerating Cell-Free DNA Fragmentation Analysis with a High-Speed Computational Toolkit

**DOI:** 10.1101/2024.05.29.596414

**Published:** 2024-06-02

**Authors:** James W. Li, Ravi Bandaru, Yaping Liu

**Affiliations:** 1. Department of Biochemistry and Molecular Genetics, Feinberg School of Medicine, Northwestern University, Chicago, IL 60611; 2. Robert H. Lurie Comprehensive Cancer Center of Northwestern University, Chicago, IL 60611; 3. Department of Computer Science, Wake Forest University, Winston-Salem, NC 27109

## Abstract

Cell-free DNA (cfDNA) fragmentation pattern represents a promising non-invasive biomarker for disease diagnosis and prognosis. Numerous fragmentation features, such as end motif and window protection score (WPS), have been characterized in cfDNA genomic sequencing. However, the analytical tools developed in these studies are often not released to the liquid biopsy community or are inefficient for genome-wide analysis in large datasets. To address this gap, we have developed FinaleToolkit, a fast and memory efficient Python package designed to generate comprehensive fragmentation features from large cfDNA genomic sequencing data. For instance, FinaleToolkit can generate genome-wide WPS features from a ~100X cfDNA whole-genome sequencing (WGS) dataset in 1.2 hours using 16 CPU cores, offering up to a ~50-fold increase in processing speed compared to original implementations in the same dataset. We have benchmarked FinaleToolkit against original studies or implementations where possible, confirming its efficacy. Furthermore, FinaleToolkit enabled the genome-wide analysis of fragmentation patterns over arbitrary genomic intervals, significantly boosting the performance for cancer early detection. FinaleToolkit is open source and thoroughly documented with both command line interface and Python application programming interface (API) to facilitate its widespread adoption and use within the research community: https://github.com/epifluidlab/FinaleToolkit

## Introduction

Circulating cell-free DNA (cfDNA) in peripheral blood has been widely utilized as a noninvasive biomarker for cancer diagnosis and prognosis^1,2^. cfDNA is characterized by highly nonuniform fragmentation across the genome, with patterns that have been linked to various epigenetic marks within the originating cells^3–9^. Numerous fragmentation pattern features have been identified in cfDNA genomic sequencing, including fragment coverage and size at transcription start sites (TSS)^3,10^ and transcription factor binding sites (TFBS)^11^, both raw and adjusted window protection scores (WPS)^4^, DNA evaluation of fragments for early interception (DELFI)^12^, end motifs, motif diversity scores (MDS)^13^, and cleavage ratios near CpG sites^7^. These patterns have been shown as promising biomarkers to boost the performance of cfDNA for disease diagnosis and prognosis^12,14^. However, source codes from these studies are often absent, poorly documented, non-executable, or not ready for the genome-wide analysis of large genomic datasets, which poses a significant barrier to the clinical application of cfDNA fragmentomics.

**Figure 1.**
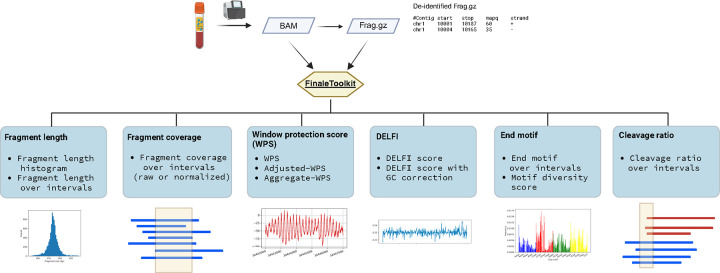
FinaleToolkit workflow. Created with BioRender.com.

Here, we introduce FinaleToolkit (Fig.1), a standalone, open-source, and thoroughly documented Python package that can efficiently extract over ten popular fragmentation patterns from a large cfDNA genomic sequencing dataset. FinaleToolkit replicates fragmentation patterns previously published, facilitating analyses when original source codes are unavailable or impractical to use. This tool supports multithreading for all the features, allowing for the processing of large genomic datasets with a speed increase of up to 50 times compared to existing implementations. Its rapid and memory-efficient design enables comprehensive genome-wide analysis of various fragmentation features across specified genomic intervals, which significantly boost the performance of cfDNA fragmentomics for cancer early detection.

## Results

**Figure 2.**
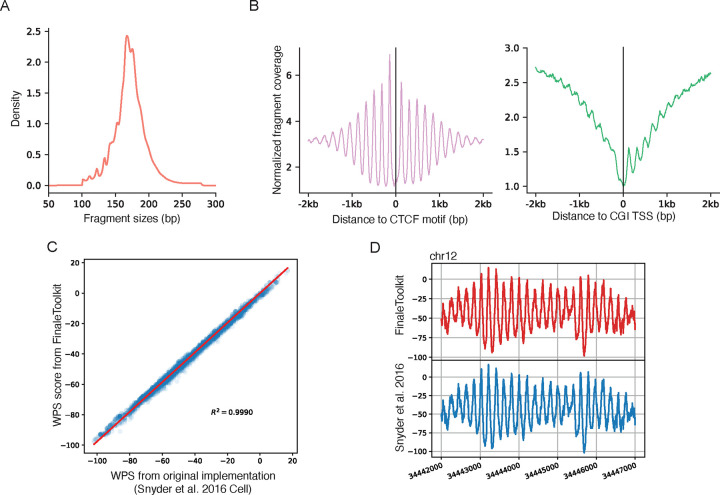
FinaleToolkit generated concordant cfDNA fragmentation patterns similar to those from the previous studies. (a). Histogram of fragment size distribution in cfDNA. (b). Normalized fragment coverages near transcriptional starting sites and CTCF motifs. (c). Scatterplot of genome-wide window protection scores (WPS) from BH01 was calculated by FinaleToolkit, and that was calculated from Snyder et al.’s 2016 Cell. Pearson correlation coefficient and p-value are calculated by the *cor.test* function in R (v4.2.2). (d). Example plot of WPS score from BH01 was calculated by FinaleToolkit (upper panel), and that was calculated from Snyder et al.’s 2016 Cell (lower panel) at a specific genomic region in chr12.

We initially employed publicly available cfDNA deep WGS data (BH01^4^, ~100X, ~1 billion fragments in total, ~168Gb bam file) from healthy and non-pregnant individuals as our primary dataset to replicate fragmentation features reported in prior studies. The genome-wide fragment length distribution aligned with expected cfDNA-specific patterns (Fig. 2a). Similarly, fragment coverage distributions near transcription start sites (TSS) and transcription factor binding sites (TFBS) exhibited the characteristic signatures associated with nucleosome phasing (Fig. 2b). Furthermore, genome-wide window protection scores (WPS) demonstrated a high correlation with results from the original implementations^4^ (Fig. 2c–d, Pearson correlation coefficient > 0.99, p-value < 2.2e^−16^).

**Figure 3.**
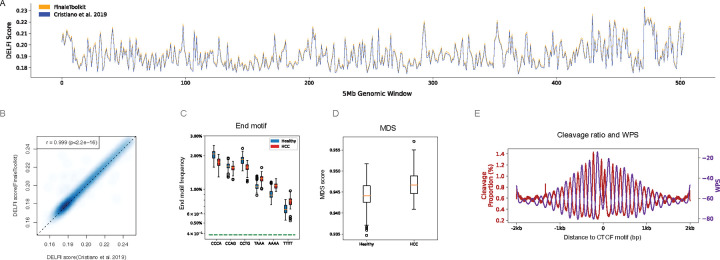
FinaleToolkit enabled genome-wide analysis of cfDNA fragmentation patterns when the original source code was not runnable or available. (a).Genome-wide DELFI score (chr1–22) generated by modified script from Cristiano et al. 2019 (blue) and FinaleToolkit (yellow) at BH01 data. (b) Scatterplot of genome-wide DELFI from BH01 calculated by FinaleToolkit and that calculated from a modified version of DELFI. Pearson correlation coefficient and p-value are calculated by the *cor.test* in R (v4.2.2). (c). End motif differences between HCC and healthy (d). Boxplot of MDS between HCC and healthy. (e). Cleavage ratio and WPS near CTCF motifs.

Due to the original DELFI source code being non-executable^12^, we benchmarked FinaleToolkit with a fork of the original DELFI scripts (see Methods in details) and achieved comparable patterns (Fig. 3a–b, Pearson correlation coefficient > 0.99, p-value < 2.2e^−16^). To replicate the differences in end motif and MDS observed between Hepatocellular carcinoma (HCC) patients and healthy individuals, we utilized publicly available cfDNA WGS data from both groups^6,12,15^. The source codes for analyzing end motif and MDS were not released in the original study. Nonetheless, our analyses using FinaleToolkit captured these fragmentation patterns effectively (Fig. 3c–d). A recent study suggested a tight correlation between the cleavage ratios near CpG sites and the DNA methylation status at CpGs^7^. Our FinaleToolkit enabled the efficient extraction of the genome-wide cleavage ratios feature from deep cfDNA WGS and observed the anti-correlated phasing patterns between nucleosomes (WPS) and cleavage ratio near CTCF, similar to the previous findings of anti-correlated phasing patterns between nucleosomes and DNA methylation patterns near the same motifs^16^(Fig. 3e). Overall, FinaleToolkit successfully demonstrated fragmentation patterns consistent with those identified in previous research.

**Figure 4.**
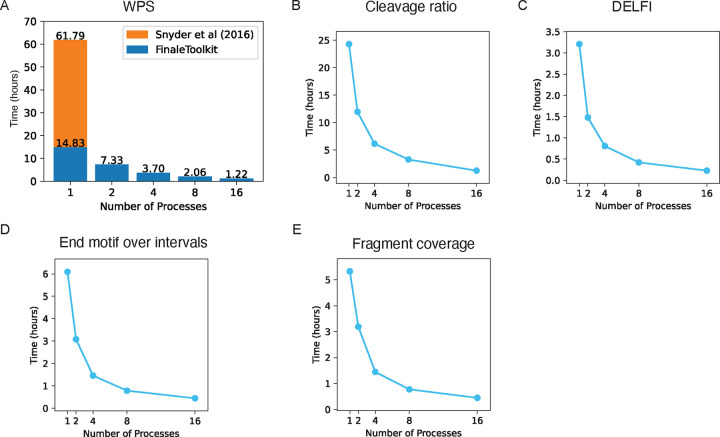
FinaleToolkit speeded up the genome-wide analysis of fragmentation patterns from large cfDNA genomic datasets. (a). Wall time cost to calculate WPS with different numbers of CPU cores. (b). Wall time cost to calculate cleavage ratio with different numbers of CPU cores. (c). Wall time cost to calculate DELFI with different numbers of CPU cores. (d). Wall time cost to calculate end motif with different numbers of CPU cores. (e). Wall time cost to calculate fragment coverage with different numbers of CPU cores.

We subsequently benchmarked the processing speed and memory usage of FinaleToolkit against the original source code when available. For the WPS study, FinaleToolkit processed genome-wide WPS features in 16.6 million windows from BH01 data in 1.22 hours using 16 CPU cores and 49 GB of memory, achieving approximately a 50-fold increase in processing speed compared to the original implementation (Fig. 4a). Remarkably, even with a single CPU core, FinaleToolkit delivered results >4 times faster than the baseline. To benchmark the DELFI script, we met memory problem with large genomic dataset even by using the fork script existed in the public domain(details in Methods). We slightly modified the public code to allow benchmark for BH01 dataset and cost ~111 Gb memory and ~4.5 hr in total. By using FinaleToolkit, we finished the whole process within 14 mins (Fig. 4c) and 1.4Gb memory(Supplementary Figure 1c), which means 19X faster with 79X less memory cost to calculate DELFI score. We also assessed the speed and memory efficiency for processing other fragmentation features in the same dataset (Fig. 4b–e). All of them can be finished within 1 hour with 16 CPUs and reasonable memory cost (Supplementary Figure 1).

**Figure 5.**
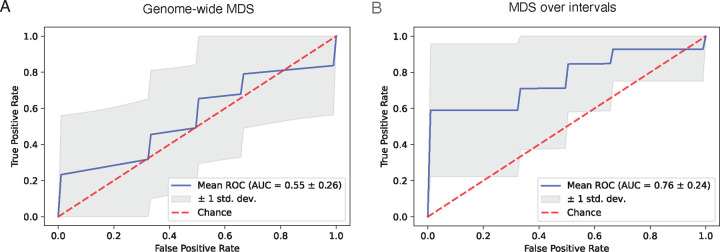
FinaleToolkit boosted the performance of cfDNA fragmentomics by genome-wide analysis of fragmentation patterns over arbitrary genomic intervals. The receiver operating characteristic (ROC) curve for distinguishing 25 early-stage breast cancer vs. 25 matched healthy controls by using (a) genome-wide MDS and (b) MDS over 100kb non-overlapped intervals. The performance is calculated by applying *xgboost* at 10-fold cross-validation and repeated ten times. For a fair comparison, (a) and (b) are compared with the same data split. The shade represents the standard deviation of ROC across repeated experiments.

Since its enhancement in efficiency, FinaleToolkit has facilitated the analysis of cfDNA fragmentation patterns across arbitrary genomic intervals, including previously unexplored measures such as end motif and MDS. Consequently, we have further demonstrated its utility in identifying aberrations in fragmentation patterns at specific genomic intervals, thereby enhancing disease diagnostic performance. A prior study identified significant differences in genome-wide summary statistics for 256 different end motifs and associated single MDS scores between early-stage cancer patients and healthy controls. We applied this strategy to our previously generated cfDNA WGS dataset from 25 matched pairs of early-stage breast cancer patients and healthy controls (matched 1:1 by age, gender, race, alcohol usage, and smoking history)^6^. Using the original approach, classification power was limited, with an Area Under the ROC Curve (AUC) of 0.55 in cross-validation (Fig. 5a, Supplementary Figure 2a). However, by applying MDS across 100kb non-overlapping intervals, we significantly improved the classification performance on the same data split, achieving an AUC of 0.76 (Fig. 5b, Supplementary Figure 2b). In summary, the advanced computational capabilities of FinaleToolkit significantly accelerate the genome-wide analysis of fragmentation patterns in large genomic datasets.

## Conclusions and Discussions

In summary, we developed FinaleToolkit to expedite the fragmentomic analysis of large-scale cfDNA genomic sequencing datasets. Current clinical applications of cfDNA fragmentomics frequently necessitate that researchers either reimplement well-known algorithms or modify existing codes for specific requirements. We have verified the efficacy of FinaleToolkit by replicating fragmentation patterns observed in prior studies or benchmarking them with the original code when available. Additionally, genome-wide summary statistics, such as end motif and MDS, ignored the variations of fragmentation patterns at different genomic regions, which may provide additional information to distinguish abnormal disease status. Our toolkit here enables genome-wide fragmentomic analysis at arbitrary genomic intervals through multithreading and boosts the performance for cancer early detection, which was not available with original software implementations for large datasets. At present, our focus is limited to popular fragmentation patterns for which source code is unavailable or unsuitable for large datasets. However, as more fragmentation patterns are identified, they will be progressively integrated into FinaleToolkit in the near future.

## Methods:

### Ethics approval and consent to participate

Not applicable.

### Data preprocessing of cfDNA WGS data

Raw sequencing datasets were processed by our previously published cfDNA workflow (https://github.com/epifluidlab/finaledb_workflow)^17^ and downloaded from FinaleDB^17^. The workflow is managed by snakemake v5.19^18^. Specifically in the workflow, raw fastq files were trimmed by Trimmomatic v0.39^19^ and then mapped to the human genome (GRCh37) using BWA-MEM 0.7.15 with default parameters^20^. Only high-quality reads were considered in the analysis (high quality: uniquely mapped, no PCR duplicates, both ends are mapped with mapping qualities more than 30 and properly paired).

### Interface of FinaleToolkit

FinaleToolkit is both a command-line program and a Python library that is accessible from the PyPI package repository using *pip*, Python’s native package manager, or *Anaconda*. This allows for easy, automated installation. Input consists of a fragmentation file (SAM, BAM, CRAM, or Frag.gz file), relevant BED, genome, and .2bit files (binary file of the reference genome, converted from fastq file by faToTwoBit from UCSC tools), and a variety of user-specified parameters. When used in the command line, files can be pipelined into and out of select commands.

### Framework of FinaleToolkit

To make FinaleToolkit more maintainable, expandable, and efficient, we implemented several functions in the *finaletoolkit.utils* module that recurs across multiple features, including functions to read one of several possible fragmentation file formats and return the fragment coordinates, functions to filter reads by flags and duplication, and functions to parse BED files.

### Benchmark WPS score

The original WPS implementation used the pipeline of three programs: an early fork of samtools to read a BAM file, a Python 2 program *FilterUniqueBam.py* to filter reads, and a Python 2 program *extractReadStartsFromBAM2Wig.py*, producing a wig.gz file containing raw WPS scores over a single interval. For benchmarking, we ran this pipeline for every 10 kb interval spanning the whole genome on a ~100x coverage file (BH01). When benchmarking FinaleToolkit, we ran *finaletoolkit wps* on BH01 over the whole genome with 1, 2, 4, 8, and 16 worker processes.

### Benchmark DELFI score

We used a fork of the original DELFI scripts (https://github.com/LudvigOlsen/delfi_scripts) due to the original DELFI implementation being highly *ad hoc* and difficult to use. This fork simplifies the command line interface, makes some optimizations on memory, and adds compatibility with BAM files aligned to GRCh38. This fork of DELFI scripts consists of 9 programs that are run in succession: *00-create_cytosine_file.r*, *00-filtered_regions.r*, *01-read_galp.r*, *02-fragment_gc.r*, *03.pre-create_bin_coordinates.r*, *03-bin_compartments.r*, *03.5-combine_bins.r*, *04–5mb_bins.r*, *06-gbm_full.r*. We ran this workflow using the “hg19” assembly option, chromosome lengths for GRCh37, and the provided gaps and filter files. We met memory problem for running *01-read_galp.r* for BH01 dataset. We had to modify the code using *reducebyYield* (from *GenomicFiles* v1.8.0 package in R), save the BAM file fragments in chunks of 1M fragments, and concatenate them, which took 95 Gb memory. It took ~110Gb memory to run *02-fragment_gc.*r and *03-bin_compartments.r* for the BH01 dataset.

To benchmark FinaleToolkit, we ran *finaletoolkit delfi* on BH01 over 100kb genome-wide bins and then merge into 5Mb window as the original study. We used the Encode Data Analysis Center blacklisted regions as a filter, UCSC Genome Browser hg19 gaps track for centromere and telomere coordinates, and used the options for GC-correction and merging bins. We ran *finaletoolkit delfi* with 1, 2, 4, 8, and 16 worker processes.

### Benchmark End Motifs and MDS

The source code was not released in the original publication^13^. To benchmark FinaleToolkit, we ran *finaletoolkit interval-end-motifs* on 10kb non-overlapped intervals or *finaletoolkit end-motifs* genome-widely at BH01 dataset with a .2bit file for GRCh37 using 1,2,4,8, and 16 worker processes. We then ran *finaletoolkit interval-mds* or *finaletoolkit mds* on the resulting tab-separated file to generate MDS data.

### Benchmark Cleavage Profiles

The source code was not released in the original publication^7^. To benchmark FinaleToolkit, we ran *finaletoolkit cleavage-profile* on BH01 data using 1,2,4,8, and 16 worker processes.

### Benchmark the speed and memory cost in FinaleToolkit

We calculated the wall time and memory cost with different numbers of CPU cores in the computational cluster (AMD EPYC 7742 CPU @ 2.0 GHz).

### Classification of early-stage cancers

We utilized our previously published cfDNA WGS dataset from 25 pairs of early-stage breast cancer and matched healthy controls^6^. We started with the de-identified fragment files from zenodo.org (breast.tar). To obtain genome-wide MDS, we first ran *finaletoolkit end-motifs* and then ran *finaletoolkit mds* on the resulting tab-separated file to generate MDS data for each sample. To obtain MDS at different genomic intervals, we ran *finaletoolkit interval-end-motifs* with default parameters on 100kb non-overlapped intervals from autosomes in hg19 and then ran *finaletoolkit interval-mds* on the resulting tab-separated file to generate MDS data for each interval in each sample. The samples were split into ten-fold cross-validation and repeated ten times by using *RepeatedStratifiedKFold* from *scikit-learn*(v1.4.2). In the end, we utilized *XGBClassifier* from *xgboost* package (v2.0.3) with the following parameters: “max_depth=5, learning_rate=0.1, n_estimators=500, objective=‘binary:logistic’,reg_alpha=0.5,reg_lambda=0.5,subsample=0.667” to calculate ROC. To make a fair comparison, we generated the same sample split for the study of genome-wide MDS and interval MDS.

We also repeated the classification on the same data split using *LogisticRegression* from *scikit-learn*(v1.4.2) with the following parameters: “penalty=‘l2’, dual=False, tol=0.0001, C=1.0, fit_intercept=True, intercept_scaling=1, class_weight=None, solver=‘lbfgs’, max_iter=100, multi_class=‘auto’, verbose=0, warm_start=False, l1_ratio=None”.

## Supplementary Material

Supplement 1

## Data Availability

The publicly available cfDNA WGS data used in this study are available in the Gene Expression Omnibus (GEO) database under accession code [BH01: GSE71378] and the European Genome–Phenome Archive (EGA) database under accession code [HCC and healthy: EGAS00001001024]. The de-identified fragment files are also available from FinaleDB and Zenodo [breast cancer and healthy (breast.tar): https://zenodo.org/records/6914806].

## References

[1] Lo Y. M. D. Cell-free DNA for Colorectal Cancer Screening. N. Engl. J. Med. 390, 1047–1050 (2024). 38477993 10.1056/NEJMe2311101

[2] Liu Y. At the dawn: cell-free DNA fragmentomics and gene regulation. Br. J. Cancer (2022). 10.1038/s41416-021-01635-z PMC8810841 34815523

[3] Ivanov M., Baranova A., Butler T., Spellman P. & Mileyko V. Non-random fragmentation patterns in circulating cell-free DNA reflect epigenetic regulation. BMC Genomics 16 Suppl 13, S1 (2015). 10.1186/1471-2164-16-S13-S1 PMC4686799 26693644

[4] Snyder M. W., Kircher M., Hill A. J., Daza R. M. & Shendure J. Cell-free DNA Comprises an In Vivo Nucleosome Footprint that Informs Its Tissues-Of-Origin. Cell 164, 57–68 (2016). 26771485 10.1016/j.cell.2015.11.050 PMC4715266

[5] Liu Y. FinaleMe: Predicting DNA methylation by the fragmentation patterns of plasma cell-free DNA. Nat. Commun. 15, 2790 (2024). 38555308 10.1038/s41467-024-47196-6 PMC10981715

[6] Zhou X. CRAG: de novo characterization of cell-free DNA fragmentation hotspots in plasma whole-genome sequencing. Genome Med. 14, 138 (2022). 36482487 10.1186/s13073-022-01141-8 PMC9733064

[7] Zhou Q. Epigenetic analysis of cell-free DNA by fragmentomic profiling. Proc. Natl. Acad. Sci. U. S. A. 119, e2209852119 (2022). 36288287 10.1073/pnas.2209852119 PMC9636966

[8] An Y. DNA methylation analysis explores the molecular basis of plasma cell-free DNA fragmentation. Nat. Commun. 14, 287 (2023). 36653380 10.1038/s41467-023-35959-6 PMC9849216

[9] Liu Y. Spatial co-fragmentation pattern of cell-free DNA recapitulates in vivo chromatin organization and identifies tissues-of-origin. BioRxiv (2019).

[10] Ulz P. Inferring expressed genes by whole-genome sequencing of plasma DNA. Nat. Genet. 48, 1273–1278 (2016). 27571261 10.1038/ng.3648

[11] Ulz P. Inference of transcription factor binding from cell-free DNA enables tumor subtype prediction and early detection. Nat. Commun. 10, 4666 (2019). 31604930 10.1038/s41467-019-12714-4 PMC6789008

[12] Cristiano S. Genome-wide cell-free DNA fragmentation in patients with cancer. Nature 570, 385–389 (2019). 31142840 10.1038/s41586-019-1272-6 PMC6774252

[13] Jiang P. Plasma DNA End-Motif Profiling as a Fragmentomic Marker in Cancer, Pregnancy, and Transplantation. Cancer Discov. 10, 664–673 (2020). 32111602 10.1158/2159-8290.CD-19-0622

[14] Foda Z. H. Detecting Liver Cancer Using Cell-Free DNA Fragmentomes. Cancer Discov. 13, 616–631 (2023). 36399356 10.1158/2159-8290.CD-22-0659 PMC9975663

[15] Jiang P. Lengthening and shortening of plasma DNA in hepatocellular carcinoma patients. Proc. Natl. Acad. Sci. U. S. A. 112, E1317–25 (2015). 25646427 10.1073/pnas.1500076112 PMC4372002

[16] Kelly T. K. Genome-wide mapping of nucleosome positioning and DNA methylation within individual DNA molecules. Genome Res. 22, 2497–2506 (2012). 22960375 10.1101/gr.143008.112 PMC3514679

[17] Zheng H., Zhu M. S. & Liu Y. FinaleDB: a browser and database of cell-free DNA fragmentation patterns. Bioinformatics 37, 2502–2503 (2021). 33258919 10.1093/bioinformatics/btaa999 PMC8388032

[18] Mölder F. Sustainable data analysis with Snakemake. F1000Res. 10, 33 (2021). 34035898 10.12688/f1000research.29032.1 PMC8114187

[19] Bolger A. M., Lohse M. & Usadel B. Trimmomatic: a flexible trimmer for Illumina sequence data. Bioinformatics 30, 2114–2120 (2014). 24695404 10.1093/bioinformatics/btu170 PMC4103590

[20] Li H. & Durbin R. Fast and accurate short read alignment with Burrows-Wheeler transform. Bioinformatics 25, 1754–1760 (2009). 19451168 10.1093/bioinformatics/btp324 PMC2705234

[21] Boerner T. J., Deems S., Furlani T. R., Knuth S. L. & Towns J. ACCESS: Advancing Innovation: NSF’s Advanced Cyberinfrastructure Coordination Ecosystem: Services & Support. in Practice and Experience in Advanced Research Computing 173–176 (Association for Computing Machinery, New York, NY, USA, 2023). doi:10.1145/3569951.3597559.

