# Supplementary material for: FinaleToolkit: Accelerating Cell-Free DNA Fragmentation Analysis with a High-Speed Computational Toolkit
Source: bioRxiv. 2024 Dec 31:2024.05.29.596414. Originally published 2024 Jun 2. Preprint. [Version 2] doi: 10.1101/2024.05.29.596414 (PMC11160763; doi:10.1101/2024.05.29.596414)
Supplement: Supplementary file 1 [file media-1.pdf]

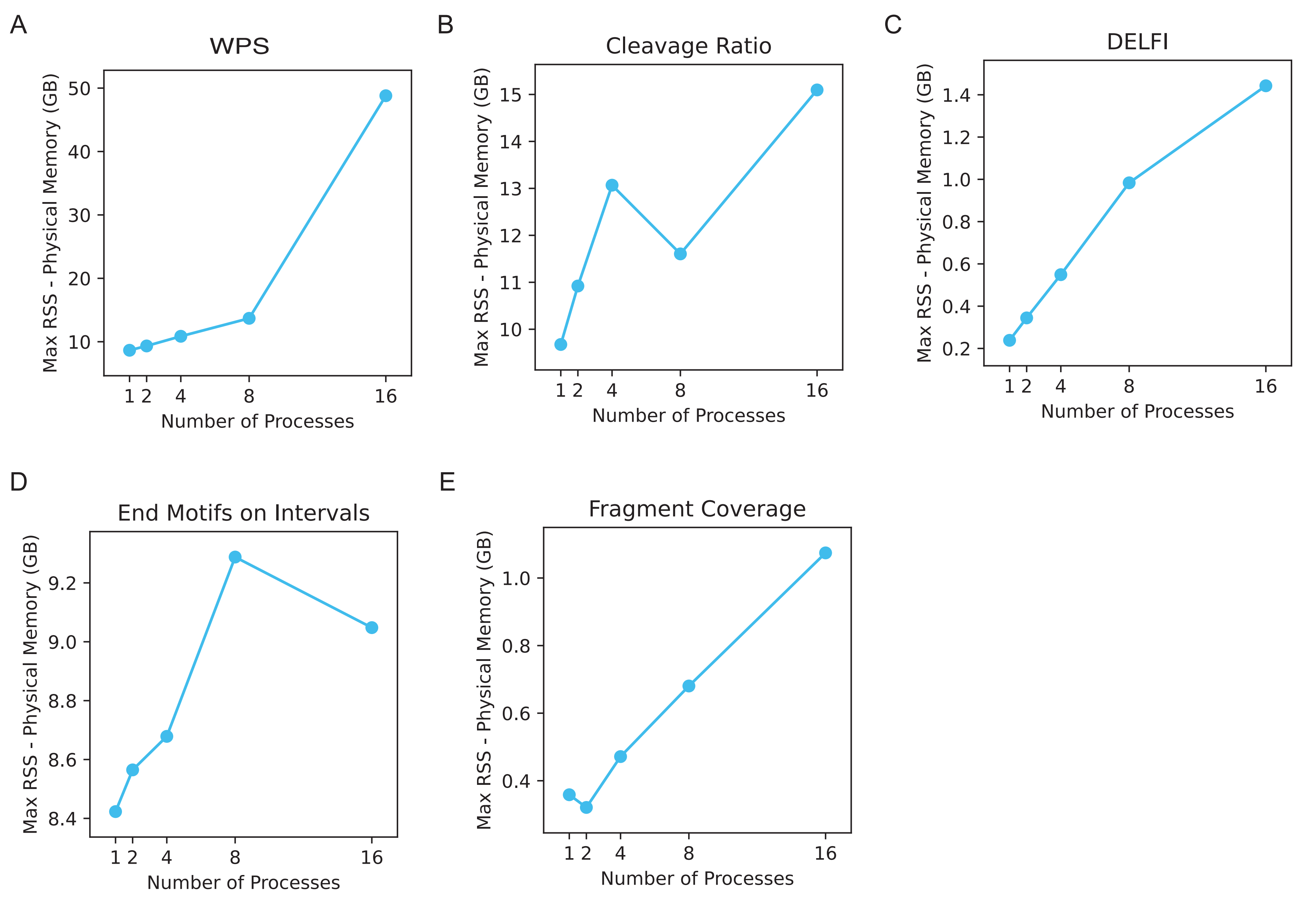
A
WPS
Max RSS - Physical Memory (GB)
10
20
30
40
50
1 2 4 8 16
Number of Processes
B
Cleavage Ratio
Max RSS - Physical Memory (GB)
10
11
12
13
14
15
1 2 4 8 16
Number of Processes
C
DELFI
Max RSS - Physical Memory (GB)
0.2
0.4
0.6
0.8
1.0
1.2
1.4
1 2 4 8 16
Number of Processes
D
End Motifs on Intervals
Max RSS - Physical Memory (GB)
8.4
8.6
8.8
9.0
9.2
1 2 4 8 16
Number of Processes
E
Fragment Coverage
Max RSS - Physical Memory (GB)
0.4
0.6
0.8
1.0
1 2 4 8 16
Number of Processes
