# Supplementary material for: FinaleToolkit: Accelerating Cell-Free DNA Fragmentation Analysis with a High-Speed Computational Toolkit
Source: bioRxiv. 2024 Dec 31:2024.05.29.596414. Originally published 2024 Jun 2. Preprint. [Version 2] doi: 10.1101/2024.05.29.596414 (PMC11160763; doi:10.1101/2024.05.29.596414)
Supplement: Supplementary file 2 [file media-2.pdf]

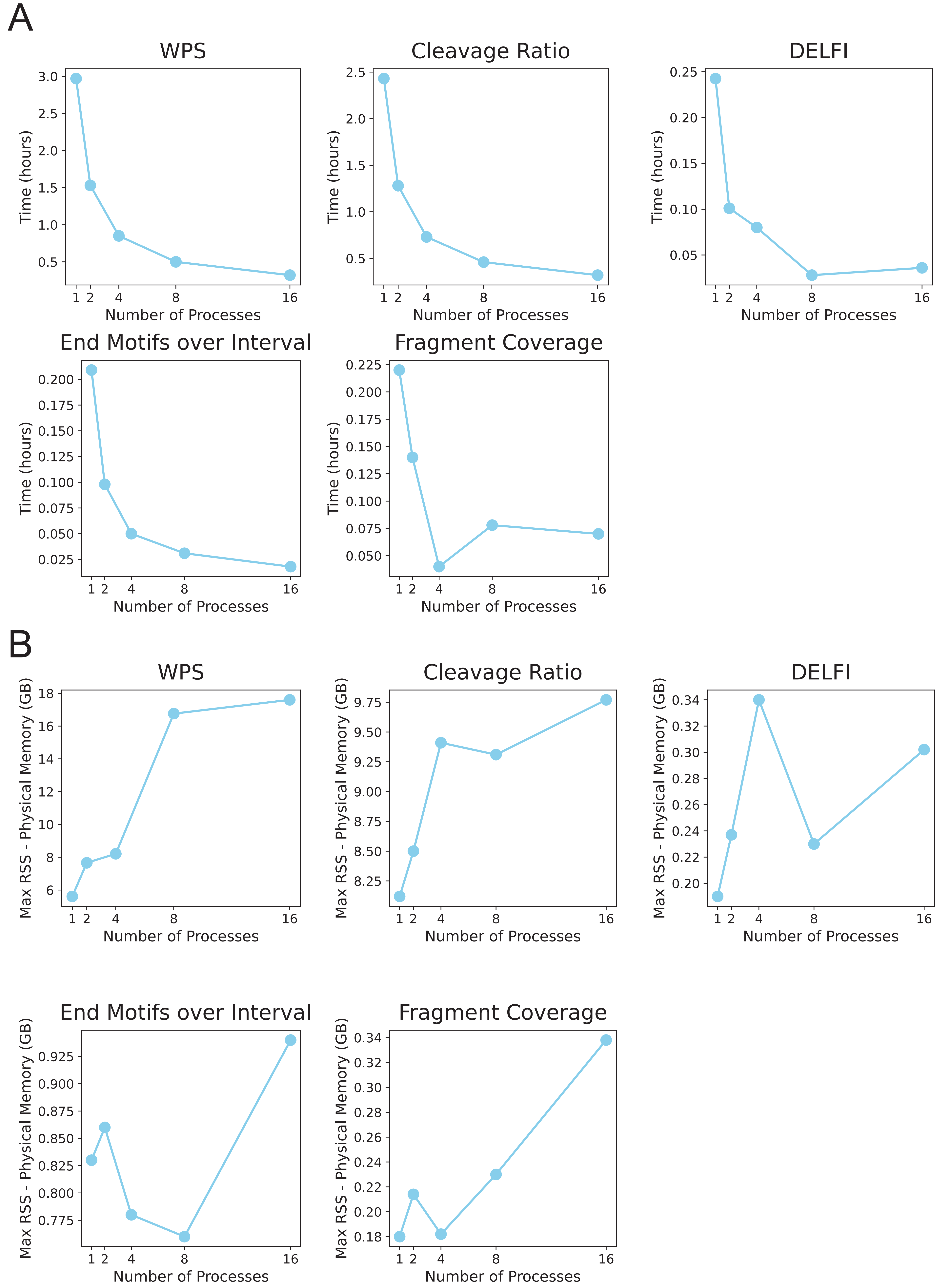
A
WPS
Cleavage Ratio
DELFI
End Motifs over Interval
Fragment Coverage
Time (hours)
Number of Processes
3.0
2.5
2.0
1.5
1.0
0.5
0.25
0.20
0.15
0.10
0.05
0.200
0.175
0.150
0.125
0.100
0.075
0.050
0.025
0.225
1 2 4 8 16
B
WPS
Cleavage Ratio
DELFI
End Motifs over Interval
Fragment Coverage
Max RSS - Physical Memory (GB)
Number of Processes
18
16
14
12
10
8
6
9.75
9.50
9.25
9.00
8.75
8.50
8.25
0.34
0.32
0.30
0.28
0.26
0.24
0.22
0.20
0.18
0.925
0.900
0.875
0.850
0.825
0.800
0.775
1 2 4 8 16
