# Supplementary material for: FinaleToolkit: Accelerating Cell-Free DNA Fragmentation Analysis with a High-Speed Computational Toolkit
Source: bioRxiv. 2024 Jun 2:2024.05.29.596414. Preprint. [Version 1] doi: 10.1101/2024.05.29.596414 (PMC11160763; doi:10.1101/2024.05.29.596414)
Supplement: Supplementary file 1 [file media-1.pdf]

FinaleToolkit: Accelerating Cell-Free DNA Fragmentation Analysis with a High-Speed Computational Toolkit

James W. Li[1-3], Ravi Bandaru[1-2], Yaping Liu[1-2,#]

Affiliations:

1. Department of Biochemistry and Molecular Genetics, Feinberg School of Medicine, Northwestern University, Chicago, IL 60611
2. Robert H. Lurie Comprehensive Cancer Center of Northwestern University, Chicago, IL 60611
3. Department of Computer Science, Wake Forest University, Winston-Salem, NC 27109

# Corresponding email: lyping1986@gmail.com

Supplementary Figures

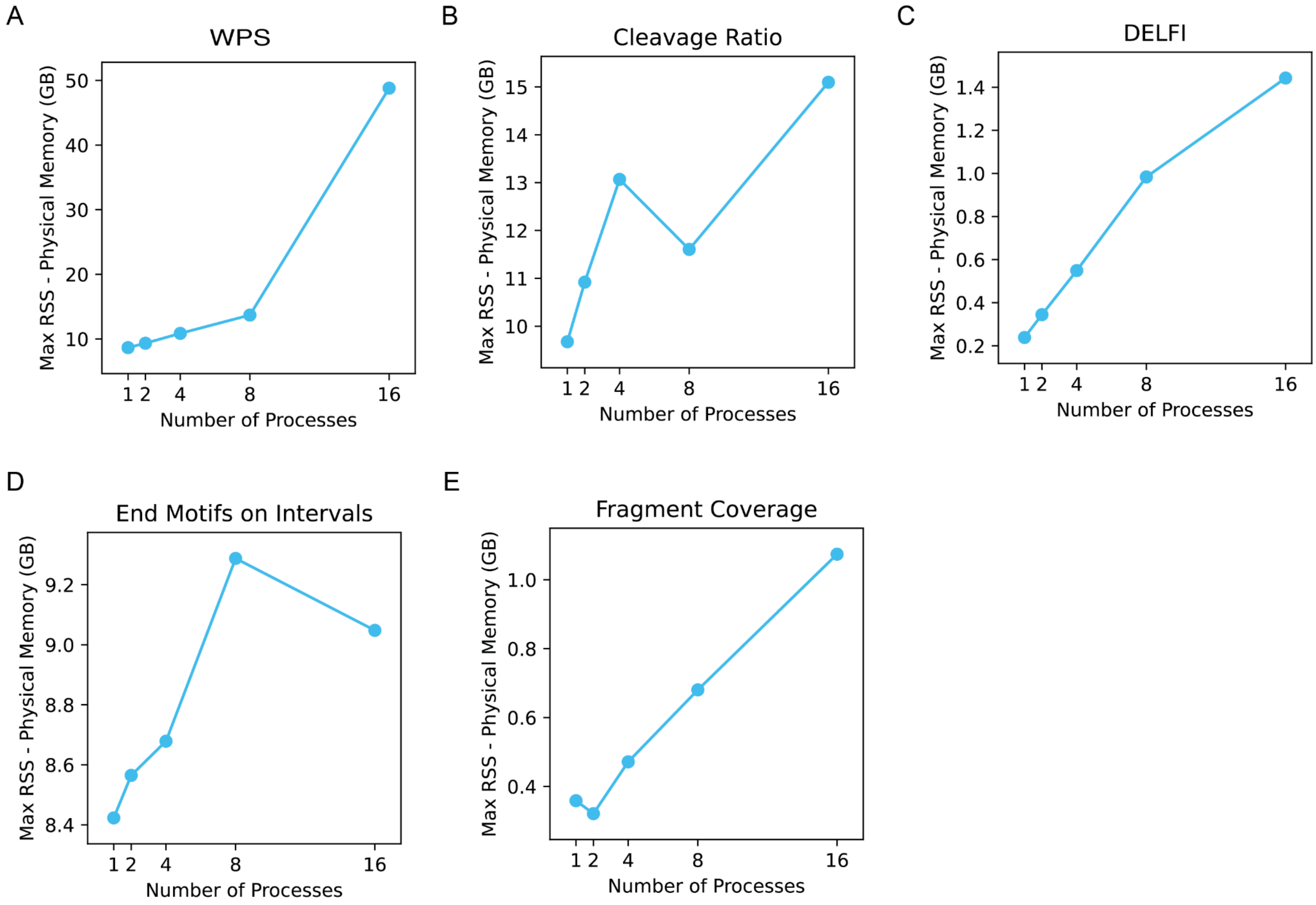

Supplementary Figure 1. Memory usage of FinaleToolkit. (a).Memory cost to calculate WPS with different numbers of CPU cores (b). Memory cost to calculate cleavage ratio with different numbers of CPU cores. (c). Memory cost to calculate DELFI with different numbers of CPU cores. (d). Memory cost to calculate end motif with different numbers of CPU cores. (e). Memory cost to calculate fragment coverage with different numbers of CPU cores.

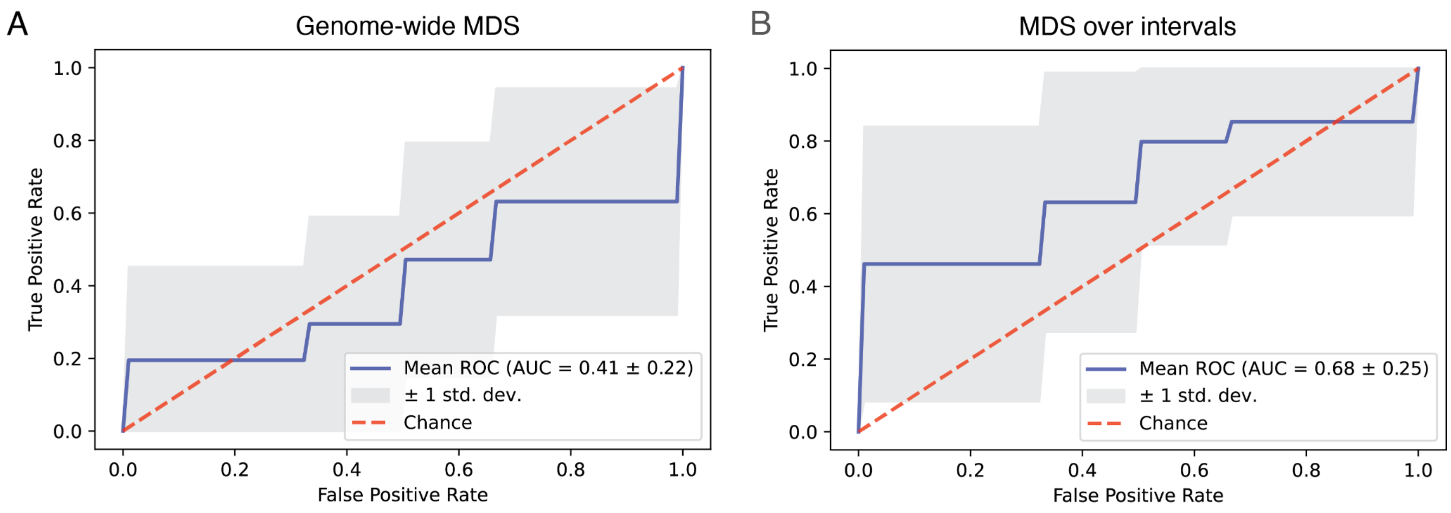

Supplementary Figure 2. FinaleToolkit boosted the performance of cfDNA fragmentomics by genome-wide analysis of MDS over arbitrary genomic intervals. The receiver operating characteristic (ROC) curve for distinguishing 25 early-stage breast cancer vs. 25 matched healthy controls by using (a) genome-wide MDS and (b) MDS over 100kb non-overlapped intervals. The performance is calculated by applying logistic regression with l2 regularization at 10-fold cross-validation and repeated ten times. For a fair comparison, (a) and (b) are compared with the same data split. The shade represents the standard deviation of ROC across repeated experiments.
